# Effects of exercise on symptoms, vestibular/ocular motor screening and postural stability in a college-aged sample

**DOI:** 10.2217/cnc-2020-0003

**Published:** 2020-05-14

**Authors:** Ryan N Moran, Nicholas G Murray, Michael R Esco, Ward Dobbs, Jamie McAllister-Deitrick

**Affiliations:** 1 Athletic Training Research Laboratory, The University of Alabama, Tuscaloosa, AL 35487, USA; 2 Neuromechanics Laboratory, University of Nevada, Reno, Reno, NV 89557, USA; 3 Exercise Physiology Laboratory, The University of Alabama, Tuscaloosa, AL 35487, USA; 4 Department of Exercise and Sport Science, University of Wisconsin-La Crosse, La Crosse, WI 54601, USA; 5 Department of Kinesiology, Coastal Carolina University, Conway, South Carolina, SC 29528, USA

**Keywords:** balance, concussion, exertion, vestibular/ocular motor screening, VOMS

## Abstract

**Aim:**

To examine the effects of maximal exercise on symptoms, vestibular/ocular motor screening (VOMS) and postural stability.

**Methodology:**

A total of 17 college-aged individuals completed a symptom scale, VOMS and the modified Clinical Test for Sensory Interaction and Balance (m-CTSIB), followed by a graded maximal exercise treadmill test. Assessments were repeated post exercise, 20 and 40 min post-exercise.

**Results:**

Significant increases in total symptoms, symptom severity scores and m-CTSIB scores from baseline to immediate post exercise were reported. Following 20-min recovery, improvements were noted on symptoms, visual motion sensitivity on VOMS and m-CTSIB.

**Conclusion:**

Symptoms and postural stability are influenced by exercise and following 20 min of rest, returned to baseline, indicating that a period of 20 min following a suspected concussion may be needed to negate exercise effects.

## Lay abstract

Baseline concussion testing is often completed by athletes in a rested state, prior to beginning their respective sports season. However, concussions are often sustained while the athlete has undergone physical exertion and is in a fatigued state. Therefore, it may be inaccurate to compare fatigue-state sideline assessments with resting-state baselines. The purpose of this study was to compare the effects of pre- and post-exercise performance on common concussion assessment tools. Worse performance was noted between pre- and immediately post-exercise on these tools. A duration of 20 min after exercise, performance returned to pretest levels, indicating that clinicians should wait at least 20 min before conducting a sideline examination after a sustained concussion, to cancel out the effects of exercise.

Recently, preseason concussion baseline testing has expanded and become prevalent in many high school and collegiate populations. In fact, the majority of certified athletic trainers in high schools (75%) and colleges (71%) reported administering preseason baseline tests [1]. Preseason concussion baseline testing is recommended over standardized norms in underrepresented or not adjusted for (i.e., learning disabilities) populations, to account for any modifying factors that may jeopardize performance outcomes [2–4]. Baseline testing allows clinicians to gauge pre-injury performance, which is primarily utilized to highlight any postinjury deficits that may help a clinician identify a concussive injury, especially considering any modifying factors or populations having a greater likelihood of below average abilities [4]. Athletes may undergo preseason baseline testing using multifaceted assessment strategies, including self-reported symptoms, neurocognitive testing, vestibular/oculomotor assessment and postural control. Often times, athletes are administered these baseline tests while they are at rest, during preseason meetings or before practices, despite concussive events routinely occurring during physical activity.

Athletes are often in a state of exertion during the on-field and sideline evaluation of a concussion. Separating exertional and physiological concussive effects on evaluative measures is essential for proper removal from play, diagnosis and management by medical professionals [5–6]. To date, exercise and exertion effects have been studied on cognition, as well as balance, which has utilized more subjective scoring systems. Covassin *et al.* [7] reported that verbal memory neurocognitive scores worsened following maximal exercise, but there were no differences on composite scores of visual memory, motor processing speed or reaction time. Retrospectively, pretest exercise on baseline neurocognitive measures yielded similar results with worse verbal and visual memory, reaction time and a greater prevalence of invalid tests [8].

Post-exercise balance measures have been investigated, indicating a greater increase in the total number of errors on the Balance Error Scoring System (BESS) following a whole-body circuit training protocol [9]. Additionally, the amount of time needed to complete a 3-m tandem gait and 10-s single-leg stance position has also been reported, with greater time post exercise [10]. Lee *et al.* [11] also reported worse BESS performance and tandem gait time following 5 min of cycling exercise. While participants in these studies completed exertional and fatigue protocols, it may be difficult to relate short term or circuit training protocol results to more in-sport demands and physiological stressors, due to greater amounts of physical exertion and neuromuscular fatigue. However, limited research is available in more recently implemented concussion assessment tests, including vestibular and ocular assessments.

To date, the only exercise effects on the vestibular/ocular motor screening (VOMS) assessment examined the level of agreement from pre- to removal from practice performance [12]. VOMS items of smooth pursuit, saccades, vertical vestibular ocular reflex (VOR) and near point of convergence (NPC) distance saw a worsening of mean scores, while convergence, horizontal VOR and visual motion sensitivity (VMS) scores improved upon removal from practice. Levels of agreement ranged from 73–89% between pre- and removal from practice. However, the time between pre- and removal measurements was approximately 2 h, which may reflect a different degree of fatigue as compared with a shorter, maximal test.

Previous methodologies have often utilized an experimental group compared with a control group, where controls may have naturally performed differently. Therefore, more controlled methodology of tracking the same individual’s pre- and post-exercise performance and recovery over time is needed. To date, very few have examined recovery timelines (e.g., 20 min of rest) following exercise testing. Covassin *et al.* [7] noted that neurocognitive test scores returned to baseline 3-days post-exercise testing. More immediate recovery times have been noted on the BESS following a circuit training based program. Specifically, BESS error performance returned to baseline, 20 min following exercise; however, Schneiders *et al.* [10] noted that single leg stance balance performance did not return to baseline levels 15-min following a bout of exercise. It remains unclear whether more objective measures of balance and postural control, as well as recently implemented vestibular/ocular motor assessments recover in similar time intervals, post-exercise [6]. Therefore, the purpose of this study was to examine the effects of maximal exercise testing and recovery timelines on symptom reporting, VOMS and postural stability. It was hypothesized that symptoms, VOMS items and postural stability would worsen post-exercise, but return to baseline levels following either a 20- or 40-min period of rest, post-exercise.

## Methodology

### Participants

A total of 17 (nine male, eight female) healthy, recreationally active, college-aged individuals (20.7 ± 2.3 years) participated in the study. Participants were recruited on campus through flyers, classes and participant referral. Any participants who had suffered a musculoskeletal injury to the lower extremity or a head injury in the 6 months prior to testing were excluded from the study. Additionally, participants were excluded for any vestibular (i.e., vertigo), visual (i.e., diplopia) or balance disorders through self-report diagnoses. Participants completed a questionnaire on their physical activity and were deemed physically fit to perform the exercise protocol in the study based on their physical activity readiness questionnaire and meeting American College of Sports Medicine (IN, USA) recommendations for physical activity.

### Measures

#### Symptom evaluation

The symptom reporting scale consisted of a 21-item list symptom checklist from the Sport Concussion Assessment Tool 5th edition (SCAT5) [5]. Each symptom was rated on a subjective scale of 0 (none) to 6 (severe). The total number of symptoms was calculated as the total number of reported symptoms greater than 0 (min = 0, max = 21). A symptom severity score was calculated as the sum of all symptom ratings (min = 0, max = 126). Further, using previously published baseline symptom factor structures [13–15] symptoms were classified into four factors: somatic (headache, pressure in head, neck pain, nausea or vomiting, dizziness, blurred vision, balance problems, sensitivity to light and noise), cognitive (feelings slowed down, feel in a fog, ‘do not feel right’, difficulty concentrating and remembering, confusion), emotional (more emotional, irritability, sadness, nervousness or anxious) and fatigue/sleep (drowsiness and fatigue/low energy). The symptom of trouble falling asleep, which is part of the SCAT5 symptom checklist, is listed as ‘if applicable’ and therefore it was not included in the study due to inapplicability to pre- and post-exercise during a single test session. The total number of symptom severity score per factor range was as follows: somatic = 0–54, cognitive = 0–36, emotional = 0–24 and fatigue/sleep = 0–12.

#### VOMS

The VOMS is a brief, clinical screening tool that assesses vestibular and ocular impairment and symptom provocation following vestibular-ocular motor tasks [16–18]. The VOMS is comprised of five item domains: smooth pursuits, horizontal and vertical saccades, convergence, horizontal and vertical VOR and VMS. Prior to administering the VOMS items, the individual is asked to rate their symptoms of headache, dizziness, nausea and fogginess on a 10-point Likert scale from 0 (none) to 10 (severe). Following the administration of each VOMS component, the athlete is then asked to rate the same four symptoms on the 0–10 Likert scale, producing a symptom provocation score. VOMS provocation scores were scored as symptom change from baseline. An objective measure, NPC distance (cm), is also recorded and averaged across three trials. The VOMS has a high overall internal consistency, with Cronbach between 0.92 and 0.97 [19,20].

#### Modified Clinical Test for Sensory Interaction & Balance

The Modified Clinical Test for Sensory Interaction and Balance (m-CTSIB) is a postural stability test conducted on the Biodex Balance System (Biodex Medical Systems, NY, USA) that assesses the sensory selection process by compromising available somatosensory, visual and vestibular senses [21]. The test provides a general assessment of how well an individual can integrate various senses and compensate when one or more of those senses are removed. The m-CTSIB consists of four different testing conditions, each assessed for 20 s, while the participant stands at the center of the balance system platform with their feet shoulder width apart and hands on their iliac crests. These conditions are: eyes open firm surface, which incorporates visual, vestibular and somatosensory inputs; eyes closed firm surface, which eliminates visual input to evaluate vestibular and somatosensory inputs; eyes open foam surface, which comprises somatosensory inputs dynamically on an unstable surface to evaluate visual and vestibular input; and eyes closed foam surface, which eliminates visual input and compromises somatosensory inputs dynamically, to evaluate vestibular input [21]. A sway index score is objectively calculated by the balance platform for each individual test condition, as well as an overall composite test score, with lower scores indicating better stability.

### Procedures

Participants completed a baseline symptom evaluation, VOMS and m-CTSIB assessment. To limit any test order effects, the order that assessments were administered was counterbalanced and remained in order through serial assessments. Following baseline assessment, participants relocated to the adjacent room to the exercise physiology laboratory and were fitted with a heart rate monitor (Polar T31; Polar Electro Oy, Kempele, Finland) at the level of the xiphoid process and informed on how to utilize the Borg (6–20) rating of perceived exertion (RPE) scale [22]. Once fitted, the participants were asked to complete a graded maximal exercise test on a motorized treadmill (TMX428CP; Trackmaster Treadmills, KS, USA). Following a brief warmup, 3-min walk at 2.7 kph and 0% grade, participants began exercising at predefined speeds and grades over 3-min stages until volitional fatigue [23]. Throughout the testing, heart rate was assessed continuously as an objective measure of exertion and RPE was acquired in the final 30 s of each stage to obtain a subjective measure of exertion. The graded exercise treadmill test was finished when the participant indicated their maximal exertion by voluntarily stopping the treadmill or indicating the investigator to stop the treadmill and the assessment ended with a brief 1-min walking cool down. At the completion of testing, heart rate and RPE were recorded as a gauge of effort and the participants were returned to the adjacent laboratory to repeat baseline measures. Upon return to the adjacent laboratory, participants were re-administered the symptom evaluation, VOMS and m-CTSIB assessments. Following the immediate post-exercise retest, participants rested and were reassessed at 20 and 40 min post-exercise, to determine if measures would recover to baseline levels [6]. Study procedures were approved by the institutional review board at the University of Alabama (AL, USA) and all participants provided consent prior to beginning the study.

### Statistical analysis

Results are presented as means and standard deviations or as percentages of the total. A series of nonparametric, Friedman tests were conducted to determine the repeated measure effects between baseline, immediate post exercise, 20 min post-exercise and 40 min post-exercise on symptoms, VOMS and postural stability performance. A Wilcoxon signed rank test was utilized to determine where differences occurred between baseline and post-exercise intervals.

## Results

### Baseline to post-exercise assessment

The average heart rate during the graded treadmill test was 192.2 ± 9.3 bpm, with an RPE of 17.8 ± 1.5. Significant differences were noted between baseline and immediate post-exercise with increases of total symptom score (p = 0.013) and symptom severity scores (p = 0.002) (Table 1). Exploration into baseline symptom factors revealed differences post exercise on somatic (p = 0.002), cognitive (p = 0.05) and fatigue/sleep (p = 0.001) scores (Table 1). No differences were reported between baseline and immediate post-exercise on emotional symptom factors (p = 0.91).

**Table 1.** Symptom reporting and factor scores at baseline and post-exercise.

Variable	Baseline	Post-exercise	20 min post	40 min post
Total symptoms^†^^,^^‡^^,^^§^	1.35 ± 1.9	4.00 ± 3.8	1.88 ± 3.1	1.00 ± 3.18
Symptom severity^†^^,^^‡^	1.53 ± 2.1	7.24 ± 6.3	2.53 ± 5.04	1.71 ± 5.5
Somatic^†^^,^^‡^^,^^§^	1.18 ± 2.0	2.59 ± 3.2	0.76 ± 1.4	0.53 ± 1.5
Cognitive^†^^,^^‡^^,^^§^	0.65 ± 1.2	1.94 ± 2.5	0.35 ± 1.0	0.24 ± 1.0
Emotional	0.76 ± 2.2	0.82 ± 2.2	0.59 ± 1.9	0.47 ± 1.9
Fatigue/sleep^†^^,^^‡^	0.41 ± 0.7	3.29 ± 1.9	0.41 ± 0.7	0.47 ± 1.2

† Significant at the 0.05 level between baseline and post-exercise.

‡ Significant at the 0.05 level between post-exercise and 20 min post-exercise.

§ Significant at the 0.05 level between baseline and 40 min post-exercise.

**Figure 1.**
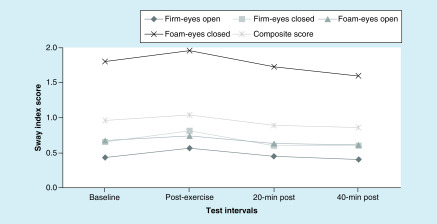
Modified Clinical Test for Sensory Interaction and Balance performance at baseline and post-exercise intervals. Mean sway index scores for individual m-CTSIB conditions and composite score at baseline, immediately post-exercise, 20 min post-exercise and 40 min post-exercise. m-CTSIB: Modified Clinical Test for Sensory Interaction and Balance.

With regard to the VOMS assessment, no differences were noted between baseline and post-exercise measures on all VOMS items and NPC distance (Table 2). Differences were observed on m-CTSIB composite score (p = 0.01) and the firm surface eyes open (p = 0.01) test condition. No differences existed on the conditions of firm surface eyes closed (p = 0.06), foam surface eyes open (p = 0.15) and foam surface eyes closed (p = 0.11) (Figure 1).

**Table 2.** Vestibular/ocular motor screening change scores at baseline and post-exercise intervals.

Variable	Baseline	Post-exercise	20 min post	40 min post
Smooth pursuits	0.12 ± 0.3	0.29 ± 0.7	0.06 ± 0.2	0.06 ± 0.2
Horizontal saccades	0.18 ± 0.5	0.29 ± 0.7	0.00 ± 0.0	0.00 ± 0.0
Vertical saccades	0.24 ± 0.5	0.41 ± 1.2	0.12 ± 0.3	0.12 ± 0.3
Convergence	0.00 ± 0.0	0.24 ± 0.6	0.12 ± 0.4	0.12 ± 0.4
Horizontal VOR	0.59 ± 0.8	0.94 ± 1.2	0.71 ± 1.2	0.47 ± 1.0
Vertical VOR	0.41 ± 1.0	0.65 ± 1.1	0.35 ± 1.0	0.35 ± 1.2
VMS^†^	0.47 ± 1.0	0.94 ± 1.0	0.29 ± 0.9	0.35 ± 0.9
NPC distance	2.78 ± 3.3	3.99 ± 7.2	3.60 ± 6.8	3.57 ± 6.9

† Significant at the 0.05 level between post-exercise and 20 min post-exercise.

NPC: Near point of convergence; VOR: Vestibular ocular reflex; VMS: Visual motion sensitivity.

### Recovery timelines

Friedman test results revealed significant findings on total symptom scores, with a significant decrease from immediate to 20 min post-exercise (p = 0.002) and 20–40 min post-exercise (p = 0.006) (Table 1). Further, significant differences were observed with lower scores at 40 min post-exercise when compared with baseline scores (p = 0.007). Similar results were noted for symptom severity scores with a decrease in scores from immediate to 20 min post-exercise (p < 0.001) and 20–40 min post-exercise (p = 0.03). No differences were observed between total symptoms (p = 0.92) and symptom severity (p = 0.75) scores between baseline and 20 min post-exercise. Significant differences were noted for the somatic symptom factor between immediate to 20 min post-exercise (p = 0.007) and baseline to 40 min post-exercise (p = 0.01), but no differences between 20 and 40 min post-exercise (p = 0.15). Similar results existed for cognitive factor scores between immediate and 20 min post-exercise scores (p = 0.01) and baseline to 40 min post-exercise (p = 0.02), but no differences between 20 and 40 min post-exercise. As expected, due to physical exertion fatigue/sleep factor revealed differences between immediate post exercise and 20 min post-exercise (p = 0.001) and between 20 and 40 min post-exercise (p = 0.03). No differences were noted between baseline and 40 min post exercise (p = 0.70). Lastly, emotional factors did not differ from post-exercise to 20 min (p = 0.18), nor from 20 to 40 min post-exercise (p = 0.15), despite surpassing baseline levels (p = 0.06).

Significant findings were noted on m-CTSIB performance over recovery time lines. Friedman tests revealed differences on the following test surfaces conditions: firm eyes open (p = 0.02) and closed (p = 0.02), foam surface eyes open (p = 0.03) and closed (p = 0.02) and composite scores (p < 0.001) (Figure 1). Specifically, improvements were noted between immediate post exercise and 20 min post exercise composite scores (p = 0.004). No differences existed on composite scores between 20 and 40 min post-exercise (p = 0.23); however, 40 min post-exercise interval produced lower scores than at baseline (p = 0.001). No differences were noted between baseline and 20 min post-exercise on all m-CTSIB conditions (Figure 1).

Recovery intervals for the VOMS revealed improved VMS scores (p = 0.004) following 20 min of recovery post-exercise (p = 0.05). No other VOMS items differed over recovery timelines (p = 0.08–0.78).

## Discussion

The results of this study suggest that following a bout of maximal exercise, symptomatology and postural stability were worse than pre-test, baseline measures. These findings support those by Lee *et al.* [11] and Patel *et al.* [15], which both reported increases in the number of total symptoms [11,15] and symptom severity scores [15] on the graded symptom checklist from the SCAT following exercise. When examining clustered symptom factors, somatic, cognitive and fatigue/sleep factors all worsened from baseline to immediate post-exercise and immediate to 20 min post-exercise. While only significantly improvements between 20 and 40 min post-exercise existed, fatigue, somatic, cognitive and emotional factors all surpassed baseline levels with improved scores at 40 min post-exercise. Symptom factor analysis further illustrates the specific type of symptoms as opposed to using total number of symptoms and symptom severity scores for all 21 items together [15].

Post-exercise effects on the m-CTSIB yielded similar result to previous literature using the BESS test, with decreased overall performance post-exercise [6,9,11]. While the BESS uses subjective, human-scoring, fatigue plays a pivotal role in establishing and controlling balance and postural stability [24–26]. Since the m-CTSIB also examines sensory integration, the results are partially supported by previous research reporting that vestibular and visual integration remained unaffected by exercise on the NeuroCom Sensory Organization Test, despite somatosensory differences [15]. This is most likely due to the localized muscular fatigue that can heavily influence lower motor neuron activation to maintain upright stance [27]. This study noted differences on the firm surface eyes open, which utilizes all three inputs (visual, vestibular and somatosensory) and a trend toward significance on the firm surface eyes closed, which utilizes vestibular and somatosensory input. Differences in results can be attributed to the NeuroCom using a dynamic platform with sway referenced support and a tilting device, as opposed to the Biodex Balance System as a stationary device using just firm and foam surfaces. While sensory integration encompasses vestibular input, the VOMS assessment utilizes symptom provocation of vestibular-related symptoms on a series of head and eye movement tasks.

To date, the only study to examine pre- to post-VOMS scores evaluated scores after removal from practice, which did not note any differences between the two time intervals, which is consistent with the results of this current study [12]. More interestingly, the VMS component of the VOMS was the only VOMS item to significantly improve from post-exercise test to 20-min follow-up. This finding may be further supported by the VMS item having an improvement in mean scores after removal from practice [12]. Worts *et al.* also found that horizontal VOR had a decrease in mean score at removal from practice, but vertical VOR did not. It may be hypothesized that the improvements could be attributed to lateral and dynamic head movement, as opposed to a fixed head with dynamic eye movement. The significant decrease in VMS symptom provocation from immediate to 20 min post-exercise could be due to adaptation to the required task. The vestibular system stores a certain amount of head movement velocity data to aid in accomplishing certain repetitive tasks [28,29]. This head reference data are stored for a certain amount of time and could enhance repeat measurements [28]. Thus, given the short time between the administrations of the VOMS, the decrease in overall symptom provocation is a direct reflection of the brain’s ability to adapt to the task and minimize negative effects such as dizziness.

This study is also believed to be the first to report these return to baseline measures for the VOMS, but also the m-CTSIB and symptoms. It has been previously noted that balance has returned to baseline following exercise, after 5 [30], 15 [10,31] and 20 min [6], while m-CTSIB composite scores were returned after 20 min of activity. While a 20-min recovery period was the earliest post-test recovery interval, it remains unclear at which exact time-point m-CTSIB composite scores returned to baseline levels. As previously stated, this is believed to be the first study to examine the effects of post-exercise recovery timelines (i.e., 20 and 40 min post-exercise on symptoms), as a major determinant of sideline concussion diagnosis and return to play. While total symptoms and symptom severity scores were returned to baseline levels after 20 min, these results provide early preliminary justification for a period of rest of up to 20 min before a sideline or private (i.e., locker room, athletic training room) evaluation is conducted to determine if an athlete has sustained a suspected concussion. Additionally, this may cause physicians, neuropsychologists and athletic trainers to further implement the VOMS assessment into clinical diagnosis, due to the lack of exercise effects, but also the VOMS’s ability to distinguish healthy controls and concussed patients [16].

Post-exercise changes on outcome measures may be influenced by hydration status, as dehydration and fluid ingestion have been reported to negatively affect cognition [32], balance [33] and symptoms [15,33], each of which play a major role in concussion diagnosis and management. Additionally, the treadmill test did not consist a maximal oxygen consumption (VO_2_ maximum) test, so it is unclear whether participants reached their VO_2_ maximum, concurrently with RPE. Some athletes and positions, such as ice hockey players or baseball/softball pitchers, may not be operating at their VO_2_ maximum in play. Regardless of dehydration or physical exertion, fatigue may be the underlying factor, which was apparent through RPE and self-reported symptoms of fatigue and drowsiness as clusters of the fatigue/sleep factor. Significant findings on baseline fatigue/sleep symptom factors were expected as the two symptoms that comprised that factor cluster were fatigue or low energy and drowsiness. Following exertion designed to fatigue the participant, it was understood that these symptoms would be elevated concurrently with RPE.

### Clinical implications

Since baseline concussion testing is primarily administered in a rested state, it may be beneficial to administer baselines tests that will be used for sideline assessment following practice or exercise, including the VOMS, King–Devick and SCAT5 (i.e., symptoms, cognition and balance). Additionally, since recovery timelines indicated that 20 min of rest was adequate for return to baseline levels, a period of rest in a locker room, evaluation room or other designated area may negate the effects of exercise to improve diagnosis and clinical decision making of sport-related concussion. One limitation to this study was that participants were exerted using a graded max treadmill test, which may not best replicate the physiological demands of sport. The treadmill testing took approximately 15 min to complete, where some sport demands have varying periods of rest and recovery. The graded treadmill test was selected to fatigue participants in a short period of time to simulate maximal fatigue that may occur in sport, as athletes are more often injured during stages of fatigue and increased training loads [34]. Heart rate measures were only collected during the graded treadmill test. It is worth noting that heart rate may factor into the decrease and return to baseline measures, so future research should consider tracking heart rate over testing intervals. The generalizability of these findings may also be limited in other levels of participation, including pediatric or high school aged individuals. Additionally, to better understand the change in symptoms, the participants were asked to rate their symptoms for how they felt at that present time of testing, rather than how they normally feel. External factors may have played a role in higher symptoms at baseline, that may have resolved upon physical activity. Lastly, hydration and nutrition levels and status were not recorded during either test sessions; however, all participants were instructed to go about their normal dietary and hydration intake on the testing day.

## Conclusion

The interpretation of on-field and sideline symptoms and postural stability should consider the effects of exertion and fatigue on performance and test interpretation. Total symptoms and severity scores and postural stability composite scores were all worse following a bout of maximal exertion testing, but returned to baseline levels 20 min later. The VMS component of the VOMS had also improved to new baseline levels after 20 min, despite no differences on the VOMS between pre- and post-test. As symptoms may vary over short-term, it is recommended that primary healthcare providers continue to utilize a multifaceted approach to baseline and post-concussion evaluation and management, with the inclusion of more objective measures. Biomarkers, such as blood and saliva, may be hopeful in aiding the ability to better diagnose and discriminate concussion’s results from other modifying factors’ (i.e., exercise) results, although extensive research is still needed.

## Future perspective

Growing implementation of multifaceted sideline assessments for concussion has been a response to consensus recommendations and development of clinical tools, such as the VOMS. However, with increasing use of symptoms, postural stability and the VOMS, data are needed to examine the effects of exercise, as athletes are likely to be concussed and undergo a sideline assessment in an exerted state. While baseline data are important to making diagnosis and management decisions, comparing rested-state baseline tests to post-exercise and exerted-state post-injury tests may not be appropriate. It is hopeful that in the future, clinicians will begin to utilize post-exercise/exertion baseline tests for assessments being used for sideline evaluation and diagnosis. Further, tests that are solely administered in a resting state, such as computerized neurocognitive testing, will not be administered on the sideline, either in their computerized version or recently developed tablet version.

**Summary points**

- Post-exercise performance on concussion assessment tools that are used for sideline assessment are key to making accurate diagnoses.
- Many tests that are used for sideline assessment are compared with resting-state baseline measures, jeopardizing the accuracy of the data comparison.
- To date, limited research has examined the effects of exercise on multifaceted assessment, but also the recovery timeline of those exercise effects.
- The present study’s results indicated that symptoms and postural stability worsened following a bout of maximal exercise testing, but returned to baseline following 20 min of rest.
- The VOMS assessment did not differ between baseline and post-exercise, further emphasizing its utility as a sideline assessment tool.
